# Adherence to the Mediterranean diet and body composition of breast-feeding mothers: the potential role of unsaturated fatty acids

**DOI:** 10.1017/jns.2021.60

**Published:** 2021-08-13

**Authors:** Chiara Tabasso, Domenica Mallardi, Ylenia Corti, Michela Perrone, Pasqua Piemontese, Nadia Liotto, Camilla Menis, Paola Roggero, Fabio Mosca

**Affiliations:** 1 Neonatal Intensive Care Unit, Fondazione IRCCS Ca’ Granda Ospedale Maggiore Policlinico, Via della Commenda 12, 20122Milan, Italy; 2 Department of Clinical Sciences and Community Health, University of Milan, Via della Commenda 19, 20122Milan, Italy

**Keywords:** Body composition, Breast-feeding, Fat mass, Maternal eating habits, Mediterranean diet, MUFAs, PUFAs, BMI, body mass index, FM, fat mass, FMI, fat mass index, MII, Italian Mediterranean Index, MD, Mediterranean diet, MUFA, monounsaturated fatty acids, PUFA, polyunsaturated fatty acids, SFA, saturated fatty acids

## Abstract

A paucity of evidence is available regarding the impact of diet's quality during pregnancy and lactation on the body composition of breast-feeding mothers. The purpose of the present study was to evaluate the association between maternal degree of adherence to the Mediterranean diet (MD) and body composition measures specifically those relating to body fat, in the lactation period. A cross-sectional study on healthy mothers of full-term babies has been conducted. At 30 ± 10 d after delivery, anthropometric measurements and body composition were assessed. A food frequency questionnaire was performed to compute the Italian Mediterranean Index (IMI) score as an index of adherence to the MD. Data related to pregnancy such as pre-pregnancy weight, gestational weight gain and morbidities were also collected. The 147 mothers included were categorised in IMI-1 (IMI score < 5; *n* 92) and IMI-2 (IMI score ≥ 5; *n* 55) groups. IMI-2 mothers showed higher daily energy, total carbohydrates, starch and fibre intakes than IMI-1. The dietary habits of IMI-2 mothers reflect the typical characteristics of MD: they consumed higher quantities of proteins and lipids of vegetal origin, higher amounts of monounsaturated and polyunsaturated fatty acids (PUFAs) and lower saturated to PUFAs ratio. The IMI-2 group showed lower absolute fat mass and fat mass index compared to IMI-1 [(20⋅2 ± 5⋅9) *v*. (22⋅9 ± 8⋅4) kg; *P* 0⋅036 and (7⋅5 ± 2⋅2) *v*. (8⋅5 ± 3⋅1) kg/m^2^; *P* 0⋅036, respectively], whereas body weight [(61⋅1 ± 8⋅0) *v*. (63⋅3 ± 9⋅2) kg] and body mass index [(22⋅4 ± 2⋅6) *v*. (23⋅3 ± 3⋅5) kg/m^2^] were similar. The degree of adherence to the MD during pregnancy and lactation is positively associated with lower maternal fat deposition in the breast-feeding period. The higher quality of dietary lipids, probably in synergy with the assumption of starchy carbohydrates and fibre, could influence maternal body fat.

## Introduction

The Mediterranean diet (MD) is characterised by high intakes of whole-grain cereals, vegetables, fruits, legumes and nuts, fish and olive oil, while low-to-moderate amounts of dairy products, red meats, sweets and red wine are contemplated. This diet regimen allows to assume low intakes of saturated fats and high amounts of monounsaturated fatty acids (MUFAs), mainly deriving from olive oil, and polyunsaturated fatty acids (PUFAs), from fish oil (*n*-3 PUFAs), and antioxidants and fibre both present in fruits and vegetables^(1,2)^. Dietary patterns and the related indexes are widely used in numerous epidemiological studies because of their realistic representation of nutritional intakes and they could be easily used to develop dietary recommendations^(3)^. Agnoli *et al.*^4^ recently developed a new Italian Mediterranean Index (IMI) to estimate adherence to the Italian MD, adapting the Greek Mediterranean Index to Italian eating behaviour. This score, ranging from 0 to 11, is based on the intake of eleven food items some typical of MD and others typical of Western dietary regimen^(4)^. Several health benefits such as longevity and chronic diseases prevention are ascribed to the adherence to the MD^(5,6)^. MD adherence also results beneficial in terms of reducing the risk of metabolic syndrome, obesity, hypertension and diabetes^(6,7)^. Aridi *et al.* have recently demonstrated that higher adherence to the MD was associated with lower total cholesterol, lower low-density lipoprotein, lower diastolic blood pressure and lower levels of dyslipidemia^(8)^. Adherence to healthy eating patterns, such as MD during pregnancy, has been widely investigated and favourable outcomes for both mother and offspring have been demonstrated, suggesting that maternal dietary patterns can influence the early life programming^(1)^. Maternal MD adherence seems to be beneficial for offspring's health both in the short and long terms, displaying a protective role against the risk of preterm delivery, small-for-gestational-age newborn, incidence of neural tube defects, asthma and allergic diseases, obesity and metabolic syndrome^(9)^. Several studies demonstrated that a poor diet quality during pregnancy increases neonatal adiposity^(10–12)^. Beneficial effects for maternal health in terms of reduction in the incidence of gestational diabetes^(13)^, gestational hypertension and pre-eclampsia^(14)^ have been demonstrated.

The impact of MD on anthropometry and body composition of breast-feeding mothers remains partially unexplored. Thus, the purpose of the present study was to evaluate the association between maternal degree of adherence to the MD and body composition measures specifically those relating to body fat, in the lactation period.

## Methods

### Study design and participants

A cross-sectional study on mothers who delivered at the authors’ Institution from January 2019 to January 2020 has been conducted. The present study was conducted according to the guidelines laid down in the Declaration of Helsinki. All mothers, at the enrolment, provided a written informed consent. The study protocol was approved by the Institution's Ethics Committee (No. 289_2017). The inclusion criteria were mothers who delivered, after a physiological pregnancy, full-term singleton babies exclusively breastfed. Unhealthy mothers, included those who regularly consumed tobacco, substances or alcohol during pregnancy and/or lactation, were considered non-eligible. Eligible mothers were enrolled during the hospital stay, immediately after the delivery. At 30 ± 10 d after the delivery, mothers were evaluated in terms of eating habits concerning the period of pregnancy and the first month of lactation, anthropometric measurements and body composition assessment. Data related to pregnancy such as pre-pregnancy weight, gestational weight gain and morbidities were also collected.

### Maternal eating habits’ evaluation

Maternal eating habits were evaluated using the European Prospective Investigation into Cancer and Nutrition food frequency questionnaire (EPIC-ffq) driven by a member of the nutritional team of authors’ Institution^(15)^. The EPIC-ffq is a validated semi-quantitative questionnaire, designed to assess the frequencies of intakes and the portion sizes of 188 food items, investigating diet over the 12 months preceding the administration. The EPISOFT software^(16)^, linking food items to Italian Food Tables^(17)^, estimates daily energy intakes and thirty-seven micro- and macronutrient intakes. Through this software, IMI was assessed to estimate the adherence to the MD. IMI is an *a priori* index, computed on the basis of an *a priori* defined Mediterranean Dietary Pattern (MDP) representative of the MD. The MDP, on the basis of which the IMI score is calculated, comprehends eleven food items: six typical ‘Mediterranean foods’ as pasta, Mediterranean vegetables, fruits, legumes, olive oil and fish; four ‘non-Mediterranean’ foods as red and processed meat, potatoes, butter and soft drinks; and the consumption of alcohol. The total IMI score ranges between 0 and 11 points, with higher scores indicating a stricter adherence. For each typical ‘Mediterranean food’, 1 point was given if the consumption was in the highest tertile of consumption's distribution, whereas 0 point was given for consumption in the lower two tertiles. For each ‘non-Mediterranean’ food, 0 point was given if the consumption was in the lowest tertile of consumption's distribution, whereas 1 point was given for consumption in the higher two tertiles. For alcohol intake up to 12 g/d, 1 point was assigned, while for abstainers and women who consumed >12 g/d, 0 point was given^(4)^.

Mothers were categorised into two groups on the basis of the IMI score: the IMI-1 group including mothers with an IMI score of <5 and IMI-2 including mothers with an IMI score of ≥5.

Energy intakes, macronutrients’ intakes and dietetic lipid profiles of the two study groups were evaluated.

### Anthropometric measurements and body composition assessment

Body weight was measured on the integrated electronic scale of the plethysmograph BOD POD (COSMED, Italy) to the nearest 0⋅01 kg, without shoes and socks and wearing a tight fitting swimsuit. Height was measured to the nearest 0⋅1 cm with a stadiometer (Seca, Hamburg, Germany) according to standardised procedures^(18)^. Maternal body composition was assessed using the air displacement plethysmography technique (ADP) (BOD POD, COSMED, Italy) following the BOD POD assessment checklist (see additional ‘BOD POD Checklist’). Mothers, fasted for at least 8 h, entered the BOD POD system wearing a tight fitting swimsuit and an acrylic bathing cap. Subject's volume was measured in an enclosed chamber, applying Boyle's gas law that relates pressure changes to the volume of air in the chamber. The body volume measurement required two tests of the duration of 50 s each. The total body volume was automatically corrected for surface area artefact using the Dubois formula for subjects with a height of >110 cm^(19)^ and for predicted thoracic gas volume^(20)^. Body density was computed from the subject's measured mass and volume and then converted into the percentage of fat mass (FM), through the BOD POD Body Composition Tracking System Software using the Siri equation for the general population^(21)^. Maternal pre-pregnancy body weight and gestational weight gain were self-reported. Body mass index (BMI) was calculated as body weight/height^2^ and expressed as kg/m^2^. Indices of height-normalised body composition for mothers were calculated as follows: a fat mass index (FMI) as FM/lenght^2^ and a fat-free mass index as FFM/lenght^2^, both expressed as kg/m^2^.

### Statistical analysis

Descriptive statistics are presented as means ± standard deviations (sd) for continuous variables, and absolute numbers and percentage for categorical variables.

Hypothesising a weight loss of 8 % attributable to the MD^(22)^ and considering the reported mean body weight (kg) of breast-feeding mothers at 1 month after delivery (64⋅5 ± 12⋅2)^(23)^, we calculated to enrol at least 132 mothers to have *α* 0⋅05, a power of 80 % and a drop out index of 20 %.

Given the normal distribution of data, differences between the two groups were evaluated using the *t*-test for the continuous variables and the *χ*^2^-test for the categorical variables. All statistical analysis was performed using SPSS software version 20 (Inc, Chicago, IL, USA), and the statistical significance level was fixed at 0⋅05.

## Results

### Maternal basic characteristics and eating habits

Among all eligible subjects, 147 mothers were included in the study. The mothers included in the study were divided into the two groups as follows: ninety-two mothers in IMI-1 and fifty-five in IMI-2. No differences in basic characteristics were found among the two study groups (Table 1).

**Table 1.** Maternal basic characteristics according to the categorisation

	IMI-1 (*N* 92)		IMI-2 (*N* 55)	
	Mean	sd	Mean	sd
Maternal age at delivery (years)	34⋅5	4⋅3	33⋅6	5
Gestational age (weeks)	39⋅3	1⋅1	39⋅3	0⋅9
Maternal height (cm)	164⋅7	6⋅8	164⋅9	6⋅5
Maternal pre-pregnancy weight (kg)	58⋅8	8⋅1	56⋅6	7⋅1
Gestational-weight gain (kg)	12⋅9	3⋅6	12⋅4	3⋅9
	*N* (%)		*N* (%)	
Parity				
Primiparae	45 (48⋅9)		30 (54⋅6)	
Secundiparae	41 (44⋅6)		23 (41⋅8)	
Pluriparae	6 (6⋅5)		2 (3⋅6)	
Vaginal delivery	63 (68⋅4)		40 (72⋅7)	
Caucasian ethnicity	85 (92⋅4)		47 (85⋅5)	
Male offspring	45 (48⋅9)		27 (49⋅1)	

From the analysis of the EPIC-ffq, differences between groups were demonstrated in terms of quantity and quality of macronutrients (Table 2).

**Table 2.** Maternal eating habits according to the categorisation

	IMI-1 (*N* 92)		IMI-2 (*N* 55)		*P-value*
	Mean	sd	Mean	sd	
Daily energy intake (kcal/d)	2260⋅1	558⋅8	2591⋅2	815⋅6	0⋅004**
Macronutrients					
Total carbohydrates (g/d)	267⋅1	81⋅1	325⋅5	131⋅8	0⋅001**
Starch (g/d)	149⋅4	55⋅3	200⋅4	118⋅5	0⋅001**
Sugars (g/d)	117⋅3	41⋅2	124⋅7	45⋅2	0⋅315
Fibre (g/d)	22⋅8	6⋅9	29⋅0	10⋅0	<0⋅001***
Total proteins (g/d)	86⋅9	22⋅0	94⋅6	30⋅7	0⋅080
Of vegetal origin (%)	31⋅7	6⋅5	39⋅6	10⋅9	<0⋅001***
Total lipids (g/d)	99⋅5	26⋅1	108⋅9	31⋅5	0⋅05
Of vegetal origin (%)	48⋅1	8⋅7	55⋅2	11⋅6	<0⋅001***
Lipids’ profile					
MUFAs (g/d)	45⋅3	12⋅0	52⋅6	16⋅5	0⋅002**
PUFAs (g/d)	12⋅5	3⋅6	14⋅3	4⋅7	0⋅011*
Linoleic acid *n*-6 (g/d)	9⋅8	3⋅2	11⋅3	3⋅9	0⋅016*
Linolenic acid *n*-3 (g/d)	1⋅0	0⋅3	1⋅2	0⋅5	0⋅009**
*n*-3/*n*-6	0⋅1	0⋅0	0⋅1	0⋅0	0⋅861
SFAs/PUFAs	0⋅1	0⋅0	0⋅1	0⋅0	0⋅861

MUFAs, monounsaturated fatty acids; PUFAs, polyunsaturated fatty acids; SFAs, saturated fatty acids.

*P < 0⋅05, **P < 0⋅01, ***P < 0⋅001. All data are expressed as means and standard deviations for continuous variables and percentages for categorical variables.

The IMI-2 group presented higher daily energy intake and higher quantities of total carbohydrates, starch and fibre compared to the IMI-1 group, whereas sugars’ intake was the same. IMI-1 and IMI-2 groups consumed comparable intakes of total proteins and lipids, even though mothers included in IMI-2 were more likely to consume higher amounts of proteins and lipids of vegetal origin than those included in IMI-1. Moreover, the IMI-2 group showed higher amounts of MUFAs, PUFAs, *n*-6 linoleic acid, *n*-3 linolenic acid and a lower ratio between saturated fatty acids (SFAs) and PUFAs compared to the IMI-1 group. No differences were found in terms of the *n*-3/*n*-6 ratio.

### Anthropometric measurements and body composition

As shown in Table 3, at 1 month post-partum mean body weight (kg) and BMI (kg/m^2^) in the two groups were not significantly different. Mothers of IMI-2 group presented significantly lower values of absolute FM (kg) and FMI (kg/m^2^) compared to those of IMI-1.

**Table 3.** Anthropometrics measurement and body composition at 1 month post-partum according to the categorisation

	IMI-1 (*N* 92)		IMI-2 (*N* 55)		*P*-value
	Mean	sd	Mean	sd	
Body weight (kg)	63⋅3	9⋅2	61⋅1	8⋅0	0⋅153
BMI	23⋅3	3⋅5	22⋅4	2⋅6	0⋅067
FM (%)	34⋅6	6⋅2	32⋅6	6⋅4	0⋅065
FM (kg)	22⋅9	8⋅4	20⋅2	5⋅9	0⋅036*
FFM (kg)	40⋅9	5⋅6	40⋅9	4⋅6	0⋅981
FMI (kg/ m^2^)	8⋅5	3⋅1	7⋅5	2⋅2	0⋅036*

FM, fat mass; FFM, fat-free mass; FMI, fat mass index.

* *P* < 0⋅05. All data are expressed as means and standard deviations for continuous variables and percentages for categorical variables.

## Discussion

We evaluated the association between the degree of adherence to the MD and body composition in breast-feeding mothers. We demonstrated that mothers with an IMI score of ≥5 have lower FMI and lower absolute FM compared to those with an IMI score of <5.

The evaluation of the dietary habits was conducted through a validated food frequency questionnaire^(15)^. This questionnaire presents a lot of advantages such as the high variety of food items considered and the possibility to better estimate the size of the portions using plates’ images. Conversely, given the complexity of its filling, it could be easy to incur in misinterpretations of the questions. For this reason, all questionnaires were driven by trained personnel and not self-reported to obtain reliable data^(24)^. To evaluate the adherence to the MD, we used the new IMI, which is a variant of the Greek score, specifically developed for the Italian population^(4,25)^. Several diet quality indexes were developed as useful tools to measure and quantify the adherence to the MD. All these indexes differ in the number and type of food items included, scoring scheme, total range of the score and type of cut-off values to assign scoring for each food item. Specifically, IMI, which considers the sum of relative values of positive and negative components, includes all food items included in most indexes and more representative of the MD. In addition, the type of cut-off considered to account for dietary intake of each food item (except that of alcohol) is based on the distribution of the dietary intake in the study population and not on fixed amount or arbitrary^(26)^. MD indexes, despite their possible limitations due to their heterogeneity in food items and scoring, are useful tools to study the role of diet in influencing the health benefits^(3,27)^.

The macronutrients’ intakes of both our study groups were consistent with those found by Pala *et al.*^(28)^, who investigated a population of Italian women, using the same food frequency questionnaire (EPIC-ffq). Energy and macronutrients’ intakes of both IMI-1 and IMI-2 are comparable to those emerged from a literature review of Davis *et al.*^(29)^ whose aim was to quantitatively define the MD by food groups and nutrients. Dietary habits of mothers included in the IMI-2 group mostly reflect the main features of the MD^(1,2)^, and indeed they consumed higher amounts of total carbohydrates, starch and fibre than the IMI-1 group. Total carbohydrates, starch and fibre's intakes of IMI-2 group were in accordance with the Reference Intake of Nutrients and Energy for Italian Population for the requirements of nutrients (LARN): differently, mothers of IMI-1 group were slightly poor in terms of carbohydrate intakes, and insufficient for fibre^(30)^.

Given the importance of the influence of dietary fats in terms of changes in body FM^(31)^, and the lack of evidences on the influence of MD in terms of maternal body composition, we focused our attention on dietary lipid profiles and fatty acids ratios. As a consequence of MD adherence, which allows the assumption of low saturated fats, high MUFAs from olive oil and PUFAs from fish and nuts^(1,2)^, the IMI-2 group presents a better dietary lipid profile. In the present study, MUFAs and PUFAs’ intakes result higher in mothers with an IMI score of ≥5, as well as the intakes of the linoleic (*n*-6) and linolenic acids (*n*-3). Increasingly, evidence has demonstrated that the unsaturated fatty acids, typical of Mediterranean foods, promote healthy blood lipid profiles, mediate blood pressure, improve insulin sensitivity and regulate glucose levels, thus displaying a cardioprotective role^(32)^. Krishnan *et al.*^(33)^, exploring the effect of dietary fatty acids composition on substrate utilisation and body weight, have demonstrated that SFAs are more obesogenic than MUFAs and PUFAs. In addition, the authors showed that the unsaturated fats seem to be more metabolically beneficial, inducing a greater diet-induced thermogenesis, energy expenditure and fat oxidation than saturated fats. Moreover, MUFAs’ metabolic benefits are greater than those of PUFAs. Interestingly, in the present study, the SFAs/PUFAs ratio is higher in IMI-1, demonstrating an imbalance in favour of SFAs, whereas the *n*-3/*n*-6 ratio is the same in the two groups. Indeed, in the IMI-1 group, the SFAs constituted 13⋅2 % of daily energy intake, which is a higher value compared to that suggested in LARN^(30)^ and to the mean MD intakes reported in the review of Davis *et al.*^(29)^. Muka *et al.*^(34)^ have shown that dietary *n*-3/*n*-6 and SFAs/PUFAs ratios were not determinant in terms of total body fat and its distribution in women. These data seem to be partially in accordance with the present findings where, despite the different maternal FM of the two groups, the *n*-3/*n*-6 ratio of dietary intake is the same, while SFAs/PUFAs is not. The higher adiposity of mothers categorised as IMI-1 could be explained by the high consumption of SFAs, associated with the lower consumption of PUFA, compared to IMI-2. In fact, SFAs have been reported to promote adiposity, particularly abdominally located^(35)^, whereas higher dietary PUFAs, displaying a fat-oxidising effect probably implicated in lower body fat retention^(36–38)^, are associated with greater FM loss in the abdominal region^(39,40)^. Finally, to better comprehend the differences in body composition, we should also consider the different food sources of *n*-3 PUFAs: health effects of PUFAs may differ with the food they are consumed in. Indeed, *n*-3 PUFAs deriving from fish products have been demonstrated to exert different beneficial effects than those deriving from vegetable foods^(41–43)^. These data seem to be consistent with the present results that showed that IMI-2 privileged the choice of vegetable origin foods. Perfilyev *et al.*^(44)^ have demonstrated that SFAs and PUFAs, in an overfeeding regimen, induce different epigenetic changes in human adipose tissue promoting the expression of different genes involved in lipid metabolism and increasing the mean degree of DNA methylations. Moreover, it should be considered the nature of IMI, which is calculated comprehending not only Mediterranean foods but also non-Mediterranean foods such as butter, soft drinks, red and processed meats and potatoes^(4)^. This implies that the lower adherence to the MD of IMI-1 mothers is due not only to a lower assumption of Mediterranean foods but also to an higher intake of non-Mediterranean foods, which are known to be highly pro-inflammatory and adipogenic being rich in sugars, saturated fats and cholesterol. Further analysis should be performed, focusing attention also on the lean compartment and dietary factors influencing it. However, an increased number of samples could bring out further associations, also relative to other body composition measures.

Furthermore, a paucity of evidence exploring the maternal body composition during breast-feeding is available. The maternal BMI of both our study groups, at 1 month after the delivery, is within the range of normality (18⋅5–24⋅9 kg/m^2^). The present results in terms of body weight are similar to those observed by Bzikowska-Jura *et al.*^(23)^ in a study conducted on forty women at 1 month after delivery, whereas absolute FM and FM% are higher in both our study groups. Differently, mothers evaluated by Gridneva *et al.*^(45)^, if compared to mothers included in the present study, show higher weight and adiposity even if they were evaluated at 2 months after the delivery. In a recent study conducted by Rabi *et al.*^(46)^ on mothers exclusively breast-feeding their 1-month babies, FM% is similar to that found in the present study, while FMI is higher. The FMI of IMI-2 group is under the cut-off value considered for the screening of the presence of metabolic syndrome in adult women^(47)^, while that of IMI-1 is slightly higher. We argue that these discrepancies could be due to the different dietary habits due to the different regional areas of the study populations involved in the studies.

Strengths of the study were the use of a validated driven food frequency questionnaire for the evaluation of the dietary habits and the use of a validated index of adherence to the MDP, specifically targeted for the Italian population. In addition, for the body composition assessment was used the ADP, which is considered a safe, quick, reliable and valid technique^(48)^. Moreover, given that it was demonstrated that the direct measurement of thoracic gas volume could be difficult in pregnant women possibly leading to biases, and that there was no warning for the immediate post-partum period, in order to prevent any related bias, we applied to the volume the correction with predicted thoracic gas volume^(49)^. Each body composition measurement and anthropometrics were performed by the same trained member of the nutritional team, and the BOD POD checklist has always been used.

Major limitations of the present study were that the study design contemplates only one study point at 1 month after delivery, and that no data regarding body composition measurements before pregnancy were available. Lastly, further insights on the possible involvement of fibre and starchy carbohydrates in weight management and body composition during breast-feeding should be evaluated.

## Conclusions

The present study suggests that the degree of adherence to the MD is associated with a preferable body composition in terms of the reduction of absolute FM and FMI in breast-feeding mothers. Furthermore, the higher degree of adherence to this dietary pattern and specifically the higher quality of lipids consumed, probably in synergy with fibre and starchy carbohydrates, may explain the preferable body composition of those mothers. Given the beneficial and widely recognised effects of MD, additional researches are needed to explore its implications through the course of the entire lactation period, not only on maternal health but also in terms of human milk composition and neonatal outcomes.

## Supplementary material

For supplementary material accompanying this paper visit https://doi.org/10.1017/jns.2021.60. click here to view supplementary material
