# Supplementary material for: Adherence to the Mediterranean diet and body composition of breast-feeding mothers: the potential role of unsaturated fatty acids
Source: J Nutr Sci. 2021 Aug 13;10:e63. doi: 10.1017/jns.2021.60 (PMC8365532; doi:10.1017/jns.2021.60)
Supplement: Supplementary file 1 [file S2048679021000604sup001.pdf]

Body Composition Checklist

This form should be completed before and during the Body Composition assessment.

Participant ____________________________

Identification Number ___________________

Date of birth _________________

Date of the visit _______________

Section 1: BODPOD device

1.	Room temperature is between 21° and 27°C	SI	NO
2.	Room humidity is between 20% and 70% relative humidity	SI	NO
3.	Doors and/or windows are closed	SI	NO
4.	Warm-up, set up 24 h before the evaluation, was successfully completed(*)	SI	NO
5.	Analyze Hardware is performed(*)	SI	NO
6.	Last weight scale calibration was performed within the previous two weeks (date________)	SI	NO
7.	Check scale is performed(*)	SI	NO
8.	Autorun is performed(*)	SI	NO
9.	Volume is performed(*)	SI	NO

- 50 L volume test for subjects > 6 years of age __
- 20 L volume test and application of infant carrier for children < 6 years of age __

*Each of these steps must be passed; whereas the failed step must be repeated.

Section 2: Preparation of the subject before the assessment

1.	Subject's data of name, date of birth, height, ethnicity have been entered	SI	NO
2.	Subject has been fasting for at least 8 hours	SI	NO
3.	Urine and stool have been passed	SI	NO
4.	Subject is free of watch, bracelets, earrings or rings	SI	NO
5.	Subject wears only a tight fitting swimsuit and an acrylic bathing cap	SI	NO

Section 3: BODPOD assessment steps

1.	Subject is weighted	SI	NO
2.	Subject entered the BODPOD and seated with its back adherent to the seat back	SI	NO
3.	Subject breathed normally, remained quiet and relaxed during the test	SI	NO
4.	At the end of measurements the subject self-reported its current physical activity level	SI	NO

Results are appropriately recorded by the researcher:

NAME/SURNAME ____________________

Signature ___________________________
